# The loss of extension test (LOE test): a new clinical sign for the anterior cruciate ligament insufficient knee

**DOI:** 10.1007/s10195-013-0238-y

**Published:** 2013-04-05

**Authors:** Massimiliano Salvi, Francesco Caputo, Giuseppe Piu, Marco Sanna, Cristina Sanna, Giuseppe Marongiu

**Affiliations:** 1 Department of Prosthetic and Sports Surgery, Casa di Cura Lay, Via S. Ignazio da Laconi, 34, 09123 Cagliari, Sardinia Italy; 2 Orthopaedic Department, University of Cagliari, Lungomare Poetto, Cagliari, Sardinia Italy

**Keywords:** Clinical diagnosis, Anterior cruciate ligament, Clinical trial, Ligament

## Abstract

**Background:**

This prospective study was created to evaluate the reliability of a new clinical test, which we termed the “loss of extension test” (LOE test). The LOE test investigates the loss of normal maximum passive extension (MPE) of the knee due to an anterior cruciate ligament tear in comparison to the normal MPE of the healthy knee.

**Materials and methods:**

The study was divided into two consecutive parts. Part 1 was designed to assess the side-to-side difference in normal MPE in a healthy population. In part 1, 100 healthy adults were enrolled. Part 2 was designed to evaluate the LOE test reliability in injured knees. In part 2, we included 196 selected patients.

**Results:**

In part 1, the average side-to-side difference in MPE in the healthy population was not statistically significant. In part 2, the overall average side-to-side difference in MPE of the injured group was 10.1 mm ± 14.1 (min −20; max 60), which was not statistically significant (*p* = 0.52). An anterior cruciate ligament (ACL) tear was found in 121 knees among 196 patients. The average side-to-side difference in MPE in the ACL-insufficient group was 16.9 mm ± 13.4 (min −20; max 60), which was statistically significant (*p* < 0.0001). The accuracy of the loss of extension test was 83.7 %, its specificity was 93.3 %, its sensitivity was 77.7 %, its positive predictive value was 95 %, and its negative predictive value was 72.2 %.

**Conclusions:**

The reliability of the LOE test is comparable to those reported in the literature for the Lachman test and dynamic tests, so the LOE test could represent a useful tool for the diagnosis of the anterior cruciate ligament insufficient knee.

## Introduction

The reliability of a clinical test (as it is with any other evaluation method) is defined by its relative accuracy, sensitivity, specificity, and its negative and positive predictive values [1]. Up until the first half of the 1970s, a clinical diagnosis of an anterior cruciate ligament (ACL) tear was only assigned based on the results of the anterior drawer test [2], which led to an extremely poor diagnostic rate [3–10]. The clinical diagnosis of an ACL tear became more accurate with the advent of the Lachman test, as described by Torg in 1976 [10], and the pivot shift test, as described by Galway in 1972 [11]. Today, the Lachman test is still considered to be the most reliable test, with the highest sensitivity and specificity levels [3–9, 12–17].

Despite its very high specificity, the pivot shift test and modifications of it that are described in the literature [18–20] have shown lower sensitivity levels, probably due to the difficulty involved in performing them [4–8, 13–17, 21, 22]. Since then, to our knowledge, there has only been one new original test leading to a clinical diagnosis of ACL tear—the “fibular head sign” of Zaid Al-duri [23]—although there have been several suggestions for modified Lachman tests that have certainly proven useful in some cases, with demonstrated sensitivity and specificity [12, 24–29].

However, the ACL-injured knee continues to be difficult to diagnose, given that common tests are difficult to perform on anxious or large patients or by small-handed clinicians [7, 12, 14, 24, 26, 28, 29]. In this blind prospective study we have evaluated the reliability of a new clinical test that we termed the “loss of extension test” (LOE test). This test is physically very easy to perform and permits the diagnosis of ACL tears.

## Materials and methods

### How the LOE test should be performed

The acronym LOE, which stands for “loss of extension,” clearly defines what the test is going to investigate: the loss of normal maximum passive extension (MPE) of a knee affected by ACL insufficiency. The MPE of the knee may be evaluated in the prone position, as described by Sachs et al. in 1989 [30], by measuring the difference between the patient’s heels. For our purpose, we modified this method by measuring the distance between the patient’s heels while the patient lies supine on a rigid orthopedic bed with both knees extended and the examiner passively extends both knees in sequence.

**Fig. 1:**
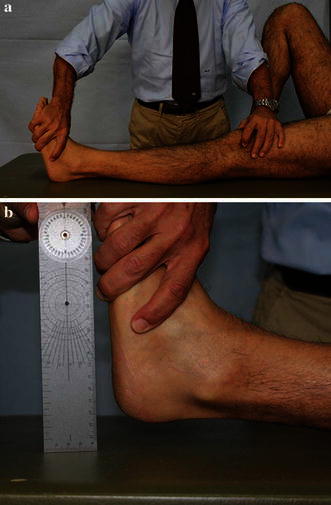
**a** The thigh of the unaffected knee is stabilized by one of the examiner’s hands, with the patellae facing forward, while the other hand extends the knee into the maximum passive extension (MPE). **b** The distance between the patient’s heel and the orthopedic bed is measured

**Fig. 2:**
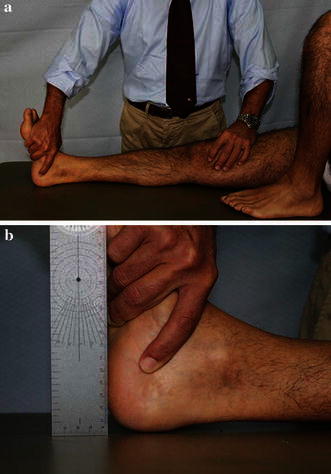
**a** The test is applied to the injured knee in the same way as described for Fig. 1. **b** The test is positive when the affected knee extends less than the healthy contralateral knee

The examiner stabilizes the thigh of the unaffected knee with one hand with the patellae facing forward, while the other hand extends the knee into the maximum passive extension (Fig. 1a). A second examiner measures the distance between the patient’s heel and the bed (Fig. 1b). The test is then applied in exactly the same way to the affected knee (Fig. 2a). The test is positive when the knee affected by an ACL tear extends less than the healthy contralateral knee (Fig. 2b).

### The LOE test’s supposed pathomechanics

Although we do not have any experimental data to prove it, we can hypothesize about the LOE test’s pathomechanics. In order to provide a possible explanation for this phenomenon, we assume that the tibia is anteriorly subluxated in extension in an ACL-insufficient knee, as previously reported by Almekinders and Chiavetta [31]. Should the tibia subluxate in extension, the posterior capsule could be abnormally tight and responsible for the limitation of the physiological maximum extension of the knee seen in cases of ACL tear. An experimental study is in progress to evaluate the tension of the posterior capsule in extension before and after ACL section.

The present study was divided into two consecutive parts. Part 1 was designed to assess the normal side-to-side difference in MPE in millimeters between the right and left knees in a healthy population. For this purpose, we enrolled one hundred healthy adults with no history, symptoms, or signs of knee pathology or injury. There were 44 (44 %) males and 56 (56 %) females. The average age was 29 years (min 16; max 44). Part 2 was designed to evaluate the LOE test reliability. For this purpose, we carried out a blind prospective study on a population of 196 new patients affected by unilateral knee pathology, before taking patient histories and before any other clinical test was applied. The affected side of the patient was also blind to the examiner.

The exclusion criteria were: patients under 15 and over 50 years of age; previous knee surgery; injured contralateral knee; loss of motion of both knees in extension. There were 158 (80.6 %) males and 38 (19.4 %) females. The average age was 30.4 years (min 15; max 50). The affected knee was the right in 100 cases (51 %) and the left in 96 cases (49 %). MRI findings, available for all of the patients (100 %), or surgical findings, available in 181 patients (92.3 %), were used as a reference standard for the final diagnosis. The level of diagnostic reliability of the LOE test was evaluated based on analyses of accuracy, sensitivity, specificity, and positive and negative predictive values, according to Levinsohn and Baker [1]. The LOE test was performed by the senior author (MS) on three consecutive occasions.

The average value obtained from the three consecutive evaluations was taken as the definite value. We arbitrarily assigned a positive value when the MPE value of the affected knee was less than that of the contralateral healthy knee, and a negative value when the opposite was true. Statistical analyses were performed using the XLSTAT 2009.5.01 software package from Microsoft. The *T* test was used for independent and pairwise samples. Linear regression analysis and the analysis of covariance model (ANCOVA) were also performed to compare different samples (pairwise comparisons were made with Bonferroni correction and the Wilcoxon test). Gender, age (for parts 1 and 2), and associated ligament injuries (for part 2 only) were statistically evaluated as independent variables. The level of significance was set at *p* < 0.05, with a confidence interval of 95 %.

## Results

### Results for part 1

The results for part 1 are reported in Table 1. The average MPE for the left knee was 35.2 mm ± 15.6 (min 0; max 70). The average MPE for the right knee was 35.7 mm ± 14.8 (min 0; max 65). The average side-to-side difference in MPE was 2.5 mm ± 5.1 (min 0; max 25). Only one case showed a side-to-side difference in MPE of more than 10 mm (25 mm; there was no clear reason for this). The side-to-side difference in MPE for healthy knees was not statistically significant (*p* = 0.79). Gender and age did not show statistical significant differences (*p* = 0.58 and *p* = 0.24, respectively).

**Table 1:** Part 1. The average difference in MPE of 2.5 mm between the right and the left knee in the healthy population was not statistically significant (*p* = 0.79)^*^

100 healthy individuals	Mean values of MPE (mm)	Standard deviation	Range (mm)
Left knee	35.2	15.6	0–70
Right knee	35.7	14.8	0–65
Side-to-side difference	2.5^*^	5.1	0–25

Based on these results, as previously reported by Portner and Pakzac [32] for other purposes, one standard deviation above the mean of the side-to-side difference in MPE of the normal population was taken as abnormal. Therefore, we considered a side-to-side difference in MPE of more than 6 mm as indicative of a positive LOE test in part 2.

### Results of Part 2

The final diagnosis is reported in Table 2. A complete ACL tear was found in 121 knees (61.7 %), whereas other knee pathologies with no ACL insufficiency were found in 75 knees (38.3 %). The ACL tear was isolated in 75 knees (61.9 %) and associated with medial or lateral collateral ligament tears in 16 knees (13.2 %). In the ACL-injured group (121 knees), there were 12 acute injuries (within 3 weeks) and 109 chronic injuries (more than 3 weeks). The average time between injury and examination was 4.9 months (min 2 weeks; max 16 months). The overall average MPE of the contralateral healthy knee was 35 mm ± 19.8 (min 5; max 105). The overall average MPE of the affected knee was 24.8 mm ± 17.1 (min 0; max 90). The overall average side-to-side difference in MPE was 10.1 mm ± 14.1 (min −20; max 60) (Table 3). The difference was not statistically significant (*p* = 0.52).

**Table 2:** Part 2. Final diagnosis for all 196 patients enrolled in part 2

Final diagnosis	*N*	%
*Isolated ACL tear*	75	38.3
*Associated ACL tear*	46	23.4
ACL + MM	25	54.2
ACL + grade 1 MCL	12	26
ACL + grade 2 MCL + MM	2	4.4
ACL + MM and LM	2	4.4
ACL + LM	2	4.4
ACL + patellar instability	1	2.2
ACL + LCL	1	2.2
ACL + grade 2 MCL + MM and LM	1	2.2
*Other knee pathology with no ACL tear*	75	38.3
MM	36	48
LM	8	10.6
Patellar tendon tendinopathy	4	5.3
Medial compartment osteoarthritis	4	5.3
Patellar instability	3	4
Anterior knee pain	3	4
Iliotibial band friction	2	2.6
Synovitis	2	2.6
Osteochondritis of the medial femoral condyle	2	2.6
Grade 2 MCL + MM	2	2.6
Grade 2 MCL	2	2.6
Osteoarthritis + MM	1	1.4
Patellofemoral osteoarthritis	1	1.4
Quadriceps tendon tendinopathy	1	1.4
Isolated LCP	1	1.4
Grade 3 chondromalacia of the patella	1	1.4
Loose body	1	1.4
Proximal tibiofibular joint sprain	1	1.4
Total	196	100

There were 75 isolated ACL ruptures and 46 associated ACL ruptures

*ACL* anterior cruciate ligament, *LCM* medial collateral ligament, *LCL* lateral collateral ligament, *PCL* posterior cruciate ligament, *MM* medial meniscus, *LM* lateral meniscus

**Table 3:** Part 2. The overall average MPE side-to-side difference of 10 mm was not statistically significant (*p* = 0.524)^*^

196 patients	Mean values of MPE (mm)	Standard deviation	Range (mm)
Healthy knee	35	19.8	5–105
Injured knee	24.8	17.1	0–90
Side-to-side difference	10.1^*^	14.1	−20 to 60

In the ACL-deficient group (121 knees), the average MPE of the healthy contralateral knee was 38.7 mm ± 21.2 (min 5; max 70) and the average MPE of the affected knee was 21.8 mm ± 16.5 (min 0; max 90). The average side-to-side difference in MPE was 16.9 mm ± 13.4 (min −20; max 60) (Table 4). The difference was statistically significant (*p* < 0.0001). Gender, age, and associated ligament tears did not statistically influence the LOE test’s reliability (*p* = 0.30, *p* = 0.80 and *p* = 0.60, respectively).

**Table 4:** Part 2. The average side-to-side difference in MPE of 16.9 mm in the ACL-deficient group (121 knees) was statistically significant (*p* < 0.0001)^*^

121 ACL-injured patients	Average MPE (mm)	Standard deviation	Range (mm)
Healthy knee	38.7	21.2	5–70
Injured knee	21.8	16.5	0–90
Side-to side-difference	16.9^*^	13.4	−20 to 60

The LOE test gave a true positive in 94 knees out of 121 with an ACL tear; it gave a true negative in 71 knees out of 75 without an ACL tear; it gave a false positive in 4 knees out of 75 without an ACL tear, and a false negative in 27 knees out of 121 with an ACL tear (3 or 25 % of acute ACL injuries and 24 or 22 % of chronic ACL injuries) (Table 5). The accuracy of the LOE test was 84.1 %, its specificity was 94.7 %, its sensitivity was 77.6 %, its positive predictive value was 95.9 %, and its negative predictive value was 72.4 % (Table 6).

**Table 5:** LOE test. True-positive, true-negative, false-positive, and false-negative cases

	No	%
True positive	94/121	77.7
True negative	71/75	94.7
False positive	4/75	5.3
False negative	27/121	22.3

**Table 6:** LOE test. Levels of sensitivity, specificity, and accuracy, as well as its positive predictive value (PPV) and negative predictive value (NPV)

Sensitivity	Specificity	Accuracy	PPV	NPV
77.7 %	94.7 %	84.1 %	95.9 %	72.4 %

## Discussion

An accurate clinical diagnosis of a pathological condition is a crucial step in medical practice before deciding on an appropriate therapeutic strategy. The same is obviously applicable to the clinical diagnosis of the ACL-insufficient knee. The aim of this prospective study was to evaluate the reliability of a new clinical test, which we termed the LOE test, for the diagnosis of an ACL-insufficient knee. The strength of this study was its prospective blind design. Clearly any information regarding patient history, the injured side, and clinical examinations could bias the examiner, when the goal of the research was to establish the accuracy of a clinical test [13, 17, 33]. Surprisingly, we found only one prospective, controlled, blind study on the accuracy of the clinical examination of ligament injuries in the literature [33].

The weakness of this study was the lack of inter-observer and intra-observer analyses between experienced and inexperienced orthopedic surgeons, which could introduce a systematic bias, and the lack of proven pathomechanics for the LOE test. However, we did not include inter-observer and intra-observer analyses because it is physically very easy for inexperienced young orthopedic surgeons and physical therapists to perform the LOE test, as we found during several years of daily practice.

The LOE test, which simply involves the passive maximum extension of the knee, has some advantages over most common tests: it does not need any expertise from the examiner, and it does not seem to be affected by either the particular conditions of the patients (grade of relaxation or size of the thigh), by the hand size of the examiner (in contrast to what has been reported for the Lachman test and dynamic tests [7, 12–14, 16, 17, 26, 33]), or by the concomitant presence of an associated tear of the medial collateral ligament, which dramatically decreases the sensitivity of the pivot shift test, as previously reported by Jonsson et al. [4], Lucie et al. [22], and Jakob et al. [21].

The lack of a validated biomechanical explanation of the pathomechanics of the LOE test is also a weakness of this study, and one that should be addressed by performing a deep clinical and experimental investigation. Nevertheless, many of the common clinical tests for an ACL tear have been described in the literature with no clear pathomechanical explanation [2, 10, 11, 18–20, 23]. This is particularly true for the Lachman test, the pivot shift phenomenon, and the fibula head sign described by Al-Duri [23]. Torg [10], in describing the Lachman test, suggested that the posterior horn of the medial meniscus could provide more false-negative cases at 70° of flexion rather than at 20° of flexion, but he did not prove it. The pathomechanics of the pivot shift phenomenon, many years after his description, are still controversial.

Recently, Claes and Bellemans, in a video on vumedi.comentitled*The Pivot Shift Unraveled. Why We Disagree with Dr Fu*, tried to explain why some ACL-insufficient knees have a large pivot shift while others do not. They stated that the reason for the pivot shift positivity is not the ACL tear itself but the ACL tear associated with the anterolateral ligament (ALL) lesion, introducing a new pathomechanical explanation of the pivot shift test. Al-Duri [23] stated that the prominence of the fibular head in ACL-insufficient knees could arise from some degree of internal rotation in such cases, but he did not prove it. At present, we can only hypothesize about the LOE test’s pathomechanics as reported above, which should be confirmed. The reliabilities of the Lachman test and dynamic tests are still controversial in the literature [3–9, 12–17, 21, 22, 25, 26, 33, 34].

The reasons for this discrepancy are probably the high heterogeneity of the population included in the studies (which makes it difficult to compare outcomes), and even more the different levels of clinical skill needed to perform them [13, 16, 17, 33]. Sholten et al. [17] reported a systematic review of the reliability of the most common diagnostic tests for the diagnosis of ACL tears, basing it on 1,090 scientific papers. Seventeen papers met the inclusion criteria established by the authors. The anterior drawer test showed a range of sensitivity from 0.18 to 0.92 (pooled sensitivity 0.62) and a range of specificity from 0.78 to 0.98 (pooled specificity 0.88). The Lachman test showed a range of sensitivity from 0.63 to 0.93 (pooled sensitivity 0.86) and a range of specificity from 0.55 to 0.99 (pooled specificity 0.91).

The pivot shift test showed a range of sensitivity from 0.18 to 0.92 and a range of specificity from 0.97 to 0.99 (pooled data were not available). Similarly, Benjaminse et al. [13] published a meta-analysis on the reliability of the same tests reported by Sholten et al. [17]. Twenty-eight scientific papers were included. They found a similar pooled sensitivity and pooled specificity for the Lachman test (0.85 and 0.94, respectively) but not for the pivot shift test and the anterior drawer test. They reported a pooled sensitivity of 0.24 for the pivot shift test and a higher pooled sensitivity and specificity for the anterior drawer test (0.92 and 0.91, respectively), even for chronic cases only. In a retrospective study on the accuracy of ACL clinical examination in a multidisciplinary sports medicine setting, after reviewing therapists, physicians, and orthopedic surgeons’ charts, Peeler et al. [16] reported only moderate levels of inter-rater reliability.

The Lachman test showed the highest level of sensitivity when administered by orthopedic surgeons (86 %), whereas it varied greatly among other clinician groups (15–87 %). This study clearly indicates that accuracy in common ACL clinical tests is very sensitive to the physician’s skill, especially in retrospective studies and non-blinded prospective ones, in which knowledge of the patient’s history and affected side emphasizes the test’s reliability [33].

The difficulty involved in performing the Lachman test and dynamic tests is well known, and is extensively described in the literature. Some modifications of the Lachman test have been introduced in an attempt to avoid false positives and false negatives. One of the most common problems leading to a false-negative Lachman test is related to the size of the clinician’s hands compared to the patient’s thigh girth [7, 12, 14, 26].

To avoid this problem, Wroble and Lindenfeld [29] introduced the “stabilized Lachman test,” in which the patient’s thigh is supported on a bolster. They reported better reproducibility of the test due to better control over tibial rotation and a fixed knee flexion angle during the examination. In 1995 [26], Draper and Schulthies described the “alternate Lachman test” as a modification of the “prone Lachman test” first introduced by Feagin [27]. They found that the sensitivity of the standard Lachman test was 28 %, that of the anterior drawer test was 59 %, while that of the alternate Lachman test was 78 % in subjects with large thigh girths (more than 43 cm). Adler et al. [12] introduced the “drop leg Lachman test,” and showed that this test was more sensitive than the standard Lachman test in bulky patients.

In order to avoid contracture in acute cases or in anxious patients, Wirth and Artmann [28] and Cross et al. [24] introduced the “active Lachman test” and the “no-touch Lachman test,” respectively, in which the examiner observes the anterior subluxation of the tibia on the femur during an active contraction of the quadriceps at 30–40° of knee flexion, without touching the patient. The Lachman test is also reported in the literature to give a false positive in cases of posterior cruciate ligament injury that cause the tibia to sag posteriorly on the femur [25, 34]. Many papers in the literature describe the reliability of common clinical tests performed on patients under general anesthesia, which contributes to the conflicting conclusions regarding the reliability of common clinical tests reported in the literature.

Katz and Fingeroth [5] reported retrospective evaluations of the reliability of the Lachman test, the anterior drawer test, and the pivot shift test in 85 patients under general anesthesia. They found 9 acute ACL tears and 13 chronic ACL tears. In the acute ACL tears, the pivot shift test was the most sensitive test (0.89), followed by the Lachman test (0.78). The anterior drawer test was the least sensitive test (0.22). In cases with chronic ACL tears, the sensitivities of the Lachman test and the pivot shift test were both 0.85, and that of the anterior drawer test was 0.54. All of the tests had specificities of more than 0.95 % in both groups. Donaldson et al. [15] found that the pivot shift test initially registered a true positive rate of only 35 %, as compared to 98 % under anesthesia, while the Lachman test was almost 100 % specific in awake patients affected by an acute ACL tear.

Similar outcomes were reported by Decker and Ruf [3] in a prospective trial including 108 patients, and by Sandberg et al. [9] and Kim and Kim [6] in two retrospective trials encompassing 182 and 147 knees, respectively. It is not surprising that the pivot shift test achieves the highest sensitivity and specificity levels under general anesthesia, even more than those of the Lachman test. Clearly, general anesthesia involves a particular condition of artificially induced relaxation that does not reflect that encountered in daily clinical practice and thus does not reproduce the test’s reliability under normal circumstances in awake patients.

To our knowledge, only one new clinical sign of the ACL-insufficient knee has been reported in the last 20 years: by Zaid Al-Duri in 1992 [23]. He reported that an abnormal prominence of the fibular head in extension was present in 100 % of 13 consecutive patients affected by ACL tears. Considering that this test was described as the most reliable test for clinically diagnosing the ACL-insufficient knee, this test is utilized surprisingly infrequently in the literature and in worldwide clinical practice. We have applied the fibular head sign in 50 consecutive documented cases of ACL tear before surgery, and we obtained a true positive rate of only 24 %.

In conclusion, the low incidence of false positives (5.3 %) implies that the LOE test has high specificity (94.7 %), very similar to that reported in the literature for the Lachman test and the pivot shift test. The relatively high incidence of false negatives (22.3 %) means that the LOE test is only fairly sensitive (77.7 %). Nevertheless, the LOE test’s sensitivity is relatively high compared to the sensitivity of the pivot shift test reported in the literature [8, 13].

Furthermore, the LOE test reliability was not affected by a concomitant medial collateral ligament injury, as described in the literature for the pivot shift test [15, 21, 22], or by the relative size of the examiner’s hands compared to the patient’s thigh girth, as described for the Lachman test [7, 12, 14, 26]. We believe that the LOE test could be a useful tool for achieving better accuracy in the diagnosis of the ACL-insufficient knee when common tests are difficult to perform on anxious patients or during the examination of large patients by small-handed clinicians. Although the LOE test could be invalidated by mechanical or painful conditions that limit knee extension, such as most acute knee injuries, it could be used and included in the routine clinical evaluation of knee injuries.
